# A Metabarcoding Analysis of the Mycobiome of Wheat Ears Across a Topographically Heterogeneous Field

**DOI:** 10.3389/fmicb.2019.02095

**Published:** 2019-09-10

**Authors:** Gabriele Schiro, Pierluigi Colangeli, Marina E. H. Müller

**Affiliations:** ^1^Leibniz Centre for Agricultural Landscape Research, Research Area 1 “Landscape Functioning”, Müncheberg, Germany; ^2^University of Potsdam, Ecology and Ecosystem Modelling, Potsdam, Germany; ^3^Berlin-Brandenburg Institute of Advanced Biodiversity Research (BBIB), Berlin, Germany

**Keywords:** Fusarium, microclimate, canopy, fungal community, Alternaria, spatially induced variance

## Abstract

Plant associated microbial communities have recently received a lot of attention because thought to play a fundamental role in plant health and development. Focusing on cultivated crops, optimized farming practices must consider the role of these communities when aiming at reducing the impact of pathogens and increasing yields. Typical inhabitants of plant’s phyllosphere are bacteria and microscopic fungi, some of them pathogenic for the plant and dangerous for the consumers, due to the production of toxins. In order to efficiently manage the microbiome, the natural drivers regulating community assembly must be clearly understood. In our study we investigated the within field variation of the phyllosphere mycobiome of wheat ears by metabarcoding of the fungal internal transcribed sequence 1 (ITS1). We selected a field characterized by a high topographic heterogeneity, which is reflected in differences in plant productivity and fitness across it. Samples were taken from 30 sampling points laid across the field where data-loggers were placed, measuring the productivity driven under canopy microclimate. The microclimatic conditions were tested as a source of potential environmental variance. Further independent spatial structures were tested using spatial eigenvector maps (MEMs). Results show considerable differences in the phyllosphere composition across the field. The local under canopy environmental conditions at each point were strong predictors of the community composition. Independent spatial effects given by the geographical position of the sampling points showed also a weaker but significant effect. Moreover we observed different spatial responses from different fungal phyla, with results resembling those described in studies done at a regional scale. This study is the first one to investigate the spatial variation of the phyllosphere mycobiome of a commercial crop within the same field. It contributes to the study of the epidemiology and community assembly dynamics of wheat phyllosphere fungi, showing how in-field community variations are the results of different environmental and spatial processes acting simultaneously. It also shows how heterogeneous fields are a smart and useful system to investigate the ecological mechanisms regulating plant microbiome composition.

## Introduction

Nowadays, modern multi-omics techniques have shed new light on the associated plant microbiota and its relationship with the host plant, giving microbes a key role in plant development, stress resistance and disease prevention (Jansson and Baker, 2016; Berg et al., 2017). Plants and their associated microbes are now described as a “holobiont,” a co-evolving unit based on symbiotic relationships between these two compartments, where microbial diversity and microbial interactions play a fundamental role in keeping the host plant healthy and productive (Vandenkoornhuyse et al., 2015; Berg et al., 2017). With a focus on cultivated plants, the need for a clearer picture of the dynamics shaping the community composition of the most common commercial crops has clearly emerged. Starting from the development of bio-control strategies, where non-pathogenic microbes are used as antagonists to pathogens, up to a more holistic view of breeding microbe-optimized plants, the microbial community of many economically important crops is nowadays being subject of studies (Singh and Trivedi, 2017; Syed Ab Rahman et al., 2018).

Among these crops is wheat (*Triticum aestivum*), which importance as worldwide staple food is out of doubt. Wheat production is threatened by various diseases, among those; fungi pose a serious menace (Figueroa et al., 2018). Common fungal diseases include blotches, caused by mainly by fungi of the family *Septoria* spp., rusts, usually associated to the genus *Puccinia* spp. or blights, like Fusarium head blight (FHB) caused by species complex involving up to 19 species, mainly belonging to genus *Fusarium* (Xu and Nicholson, 2009; Bockus et al., 2010). FHB fungi infect the ear, the grain bearing part of the plant. Along with pathogens, wheat ears are colonized by a wide variety of other fungi, considered less-pathogenic or non-pathogenic, such as *Alternaria* spp., *Cladosporium* spp. or *Epicoccum* spp. (Legard et al., 1994; Nicolaisen et al., 2014; Bankina et al., 2017). From an agricultural perspective, these phyllosphere organisms are of concern due to the caused productivity loss and for food security. Certain fungi, such as *Fusarium* or *Alternaria*, synthesize mycotoxins, chemical compounds with toxic effects for the end consumers (Bottalico and Perrone, 2002; Ostry, 2008; Vučković et al., 2012).

Studying the community assembly is useful for the manipulation of the phyllosphere community in order to control pathogenic populations. Previous studies have shown good potential in suppressing pathogens through the inoculation of antagonistic strains of fungi or bacteria (Schisler et al., 2002; Xue et al., 2014; Baffoni et al., 2015; Palazzini et al., 2016; El-Gremi et al., 2017). Fungal interactions seem to play a role also in causing differences in diseases symptoms and effects; e.g., mycotoxin accumulation has been shown to be influenced by the co-cultivation of different fungi in the laboratory (Korn et al., 2014; Siou et al., 2015). To further develop such concepts, it is important to understand which factors naturally influence the phyllosphere community. Such research should be contextualized in relation to potential variations that different environmental conditions, plant genotypes, physiologies, or agricultural practices could cause on the community composition. An efficient development of biocontrol strategies must start from a clear understanding of the community assembly dynamics, where the factors influencing the community variations are clearly individuated and accounted for their effects.

Recent developments in next generation DNA sequencing has emerged as a powerful tool for microbial ecologists, who started applying metabarcoding techniques to investigate the community associated to many organisms (Abdelfattah et al., 2018). To our knowledge, the first study applying such method on wheat was published in 2014 and analyzed the mycobiome of 90 wheat grain samples collected all over Denmark (Nicolaisen et al., 2014). The authors have found a “core” of operational taxonomic units representing 99% of all sequences with significant co-existence patterns among them. Studies from Karlsson et al. (2014, 2017) examined the effect of fungicide application and managing practices (organic and traditional agriculture) of samples of wheat leaves coming from different fields in Sweden. Sapkota et al. (2015), observed the effects of fungicide application host plant genotypes on the community composition. Hertz et al. (2016) observed the evolution of the mycobiome of wheat ears, along a timeline of ear development. Other investigations examined both the fungal and the bacterial community on different plants compartments. Granzow et al. (2017) examined the variation of the communities in a pot experiment simulating different crop regimes. Gdanetz and Trail (2017) used field samples, exploring the influence of field management practices on the wheat microbiome. All these studies observed variation in the microbial community, indicating how complex are the dynamics influencing the phyllosphere composition, as being affected by multiple factors simultaneously.

Many studies have also addressed the in-field epidemiology of important fungal pathogens. Taking *Fusarium* as an example, plenty of data is available with a focus on its spore dispersal and aerobiology (Del Ponte et al., 2003; Paul et al., 2004; Keller et al., 2014; Schiro et al., 2018). Nevertheless, to our knowledge, no study has so far investigated the spatial variation of the phyllosphere communities within a single field. Such an investigation could reveal spatially dependent structures hiding behind the mechanism of phyllosphere community assembly. Typically, spatial structures of ecological communities can be related to two fundamental processes: first, community variations could originate by differences in the environment across the sampled area. In this case, the spatial variation is told to be “induced” because the environment influences the community structure at each point. Secondly, spatial structures can be created by biotic processes generated by the community itself, such as dispersal and competition dynamics: These processes are observable through the analysis of the spatial autocorrelation among neighbor points; with closer points having a more similar community composition than those far apart. We will refer to it as “un-induced” spatial variance (Dray et al., 2006; Soininen, 2016).

In our study we investigated the spatial structure of the ear fungal community across a topographically heterogeneous wheat field. This approach allows us to reduce the number of potentially influential variables (such as plant variety and management practices), while relying on in-field differences related to the topographical diversity of the field as source of induced spatial variance. The heterogeneous topography, with hilltops and depressions, causes differences in soil type, soil moisture and plant fitness, which reflect in a spatial variations of productivity. Under-canopy microclimate (air temperature, humidity and soil moisture) is strongly connected to in-field productivity, since more biomass is responsible for more canopy cover effect (as explained later in the results section). We used this sampling method in previous studies, where we observed the variation in mycotoxins, spore deposition and genetic abundance of the genera *Fusarium* and *Alternaria* to be related to our explanatory microclimatic variables (Müller and Korn, 2013; Schiro et al., 2018, 2019). Moreover, due to the fairly regular sampling design, a single field represents a good system to test for the effect of the un-induced spatial variance, or in other words, to detect spatially explicit distributional patterns independent of the environmental variables considered.

We collected samples from 30 points scattered across a wheat field, selected for its high heterogeneity, situated in the north east of Germany. Sampling was conducted in summer 2017, 2 weeks before harvest. Via metabarcoding of the fungal internal transcribed region 1 (ITS1), we obtained a picture of the ear fungal community at each point. We here provide for the first time for a quantification of the variations of the ear phyllosphere mycobiome within the same commercially cultivated field, shedding light on its in-field biogeography. We believe that future developments of this approach will be useful for investigating how dispersal and colonization dynamics influence community assembly.

## Materials and Methods

### Field Work

**FIGURE 1:**
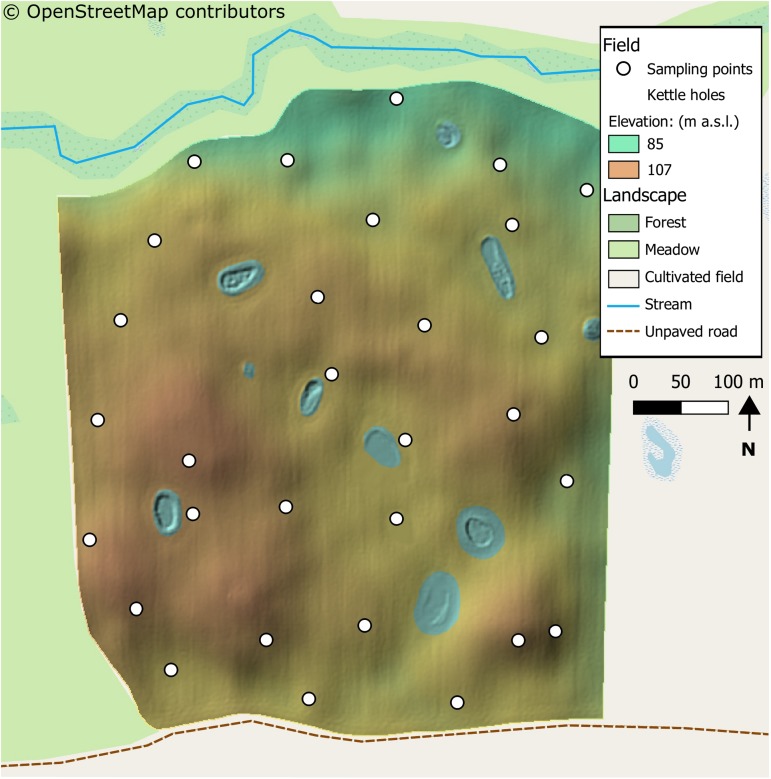
Map of the sampled field and immediate surroundings. Within the field, a digital elevation model is shown with the resolution of one meter. The landscape features around the field are also shown, as described in the legend. The landscape details are obtained from OpenStreetMap contributors (2017). Noticeable is the heterogeneity of the field, with many hilltops depressions and kettle-holes across it. The field had also heterogeneous surroundings, with a meadow, a small river (or stream) and an unpaved road on its sides.

The selected field was already used in a previous work (Schiro et al., 2018). A topographically heterogeneous field located within the AgroScapeLab Quillow (Agricultural Landscape Laboratory Quillow^1^), precisely in the proximity of the village of “Raakow, Brandenburg) in the North eastern German lowlands. The field was selected for its pronounced topographical heterogeneity and for the differences in landscape elements surrounding it. The field bordered with a forest at its northern edge, with an uncultivated meadow at its western edge, with an unpaved road and another cultivated field at its southern edge and a cultivated field at its eastern edge. The two fields bordering at the southern and eastern edge were also cultivated with wheat. The study site features small scale topographic variations, as a result of differences in Pleistocene glacial landforms (Figure 1). The field was cultivated for commercial purposes and samples were taken in agreement with the farmer. The wheat cultivar “Julius” (susceptibility to FHB is “5” in a scale from 1 to 9 (Federal Office of Plant Varieties, 2017) was grown, with maize as preceding crop. A conservation tillage method with disc cultivator less than 0.15 m deep was used. Further details on field management are presented in Supplementary Table S1. Weather data was measured by the climatic station located in the research station “Dedelow” of the Leibnitz Centre for Agricultural Landscape Research (ZALF), situated circa six km away from the research field.

Thirty sampling points were laid across the field; at each of them a set of microclimatic sensors was positioned. Sensors measured the following parameters: air temperature and humidity, soil water content and leaf wetness index (LWI). Sensors consisted of a data logger “HOBO H21-USB” recording every hour the values measured by air temperature/humidity sensor “S-THB-M002” mounted in a solar radiation shield “RS3”, a soil humidity sensor “S-SMD-M005,” and a leaf wetness sensor “S-LWA-M003,” all of them provided by Onset Computer Corporation (Bourne, MA, USA). Air temperature/humidity sensor and LWI were measured at a height of 30 cm from the ground. The experiment took place in June and July 2017. The microclimatic stations started to record values 3 weeks before the sampling date (Zadoks scale ± 73 until 90), recording a measurement every hour. At each point, in the square meter around it, the height of 15 random plants was measured. The average height per point was then used for further analysis. At each point, 15 ears were randomly picked from the plants at growth stage ±90 (Zadoks et al., 1974). The ears were placed in clean paper bags and transported to the laboratory.

### DNA Extraction

DNA extraction followed the protocol already used in Schiro et al. (2019). The 15 collected ears per each point were dried at 60°C for 48 h. Once dried, they were milled using a laboratory ball-mill MM200 (Retsch, Haan, Germany) at 1000 rpm for 45 s. The milled material was carefully mixed. 200 mg of milled plant material were put in a centrifuge tube with 100 μg proteinase K (article nr. 7528.2, Carl Roth, Karlsruhe, Germany) and 1.2 mL CTAB precipitation buffer [20 g L^–1^ CTAB (article no. 9161.3, Carl Roth), 1.4 mol L^–1^ NaCl (article no. 33614, Merck, Darmstadt, Germany), 0.1 mol L^–1^ TRIS (article no. 37180, Serva, Heidelberg, Germany) and 20 mmol L^–1^ Na2EDTA (article no. 8043, Carl Roth)] and incubated in an incubator “Enviro-genie” (Scientific Industries Inc., Bohemia, NY, United States), overnight at 65°C, with 0.5 rotation s s^–1^. After centrifugation at 10,000 g for 10 min, the supernatant was transferred to another tube and with 400 μL of chloroform (article nr. 102445, Merck). Samples were hand shaken for 30 s and then centrifuged at 12,000 g for 10 min. Circa 600 μL of the superior phase were transferred into a tube, where a double volume of CTAB precipitation buffer was added. Tubes were left resting for 1 h at room temperature and before being centrifuged at 12,000 g for 10 min. After centrifugation, the supernatant was discharged and the pellet resuspended in 350 μL NaCl 1.2 mol L^–1^, 400 μL of chloroform were added and samples were softly hand shaken for 30 s. The superior phase was transferred to a new tube and 300 μL 4°C cold isopropanol (article no. 109634, Merck) were added. Samples were incubated at 4°C for 20 min before being centrifuged for 15 min at 12,000 g. Supernatant was discharged and the pellets were additionally washed with 500 μL 70% ethanol solution (article nr. 111727, Merck). After centrifugation for 15 min at 12,000 × g, ethanol was discharged and samples were dried with a “Speedvac DNA 110” (Thermo Fisher Scientific, Waltham, MA, United States). Pellets were dissolved in 100 μL distilled sterile water and stored at −18°C until further analysis.

### PCR Amplification and Amplicon Sequencing

PCR amplification and amplicon sequencing were performed by LGC Genomics (Berlin, Germany). The PCRs included about 5 ng of DNA extract, 15 pmol of each forward primer ITS1F 5′-NNNNNNNNNNTCTTGGTCATTTAGAGGAAGTAA and reverse primer ITS2R 5′-NNNNNNNNNNTGCTGCGTTCTTC ATCGATGC in 20 uL volume of 1x MyTaq buffer containing 1.5 units MyTaq DNA polymerase (Bioline) and 2 μl of BioStabII PCR Enhancer (Sigma). For each sample, the forward and reverse primers had the same 10-nt barcode sequence. PCRs were carried out for 35 cycles using the following parameters: 2 min 96°C pre-denaturation; 96°C for 15 s, 50°C for 30 s, 70°C for 90 s. DNA concentration of amplicons of interest was determined by gel electrophoresis. About 20 ng amplicon DNA of each sample were pooled for up to 48 samples carrying different barcodes. The amplicon pools were purified with one volume AMPure XP beads (Agencourt) to remove primer dimers and other small mispriming products, followed by an additional purification on MinElute columns (Qiagen). About 100 ng of each purified amplicon pool DNA was used to construct Illumina libraries using the Ovation Rapid DR Multiplex System 1-96 (NuGEN). Illumina libraries were pooled and size selected by preparative gel electrophoresis. Sequencing was done on an Illumina MiSeq using V3 Chemistry (Illumina).

### Bioinformatics

Bioinformatics analyses were performed in R software (R Core Team, 2017). Sequences have been deposited in the NCBI Sequence Read Archive, with the BioProject ID “PRJNA517107.” Reads have been previously demultiplexed and barcodes removed by the sequencing provider. A clustering-free Divisive Amplicon Denoising Algorithm (DADA2) was used. This algorithm infers error models which then use to derive amplicon sequencing variants (ASVs). ASVs are not based on arbitrary thresholds, as in the case of defining different operational taxonomic units, therefore can detect differences in sequences down to a definition of a single nucleotide (Callahan et al., 2016, 2017). Primers were removed using the Unix command line tool cutadapt (Martin, 2011). We truncated the reads at the first quality score lower or equal to two. As output, an ASV count table, which reported the number of times each ASV has been counted per point, was produced. As Supplementary Material, the R script used as pipeline is provided. Taxonomy was assigned using the function within the DADA2 R package “assignTaxonomy” and the Version 01.12.2017 of the UNITE general FASTA release (UNITE Community, 2017). For the 20 most abundant ASVs, a blast search in the gen-bank database was also performed (Benson, 2004). Uncultured results were not considered as well as singletons. Considering these parameters, the affiliation was given to the lowest possible level.

### Statistical Analyses

Statistics was also performed using the software R (R Core Team, 2017). Due to technical problems with the data loggers, which went off during the experiment, two sampling points were excluded from the analyses. The hourly recorded values from the data loggers were averaged and scaled (with average as zero and standard deviation as unit) prior to analysis. To avoid multicollinearity between the explanatory variables, a correlation matrix was used to exclude these strongly correlated (Pearson *r* ≤ 0.9). The ASV count table was Hellinger transformed; distance-based redundancy analysis (db-RDA) was performed to test the relationship between community variation represented as a Bray-Curtis dissimilarity matrix and environmental variables (Legendre and Gallagher, 2001; Borcard et al., 2018). Automatic stepwise model building for constrained ordination methods was used to select the variables; model building was run in forward direction. These operations were done using the “vegan” package in R (Oksanen et al., 2018). Spatial data was handled in R with the package “sp.” (Pebesma and Bivand, 2005; Bivand et al., 2013). A spatial weighting matrix was constructed using the R package “spdep” (Bivand and Piras, 2015), row standardized (sums over all links to *n*), with points closer than 125 m as neighbors. This threshold was selected because it provided is the first value (at 5 m resolution), able to connect all sampling points with at least one neighbor. Moran’s Eigenvector Maps (MEMs) were generated with the package “adespatial” (Dray et al., 2018). To select statistically significant MEMs, an automatic stepwise selection method as described before was used. To partition the variation of the community data between the two explanatory matrixes we used the fuction varpart of the package “vegan” (Oksanen et al., 2018) while to test their significance, a type II Anova analysis of variance was used. A negative adjusted R squared value (−0.04) for the shared fraction appeared. We corrected the negative value according to Legendre et al. (2012) using proportional apportioning. For clustering, the counting of the 20 most abundant ASVs at the different sampling points was used. Values were scaled in order to avoid biases given by the highly different abundances among ASVs. Clustering was done using Ward method using a dissimilarity matrix of Euclidean distances. The optimal number of clusters was calculated with an average silhouette method, using the package “factoextra” (Kassambara and Mundt, 2017). In order to test the effect of the two previously selected variables on the 20 most abundant ASVs, an RDA was used. Relative abundances were also in this case standardized in order to obtain a more balanced ordination. The R script for the analysis is attached as Supplementary Material.

## Results

### Overview of the Climatic and Microclimatic Measurements

**FIGURE 2:**
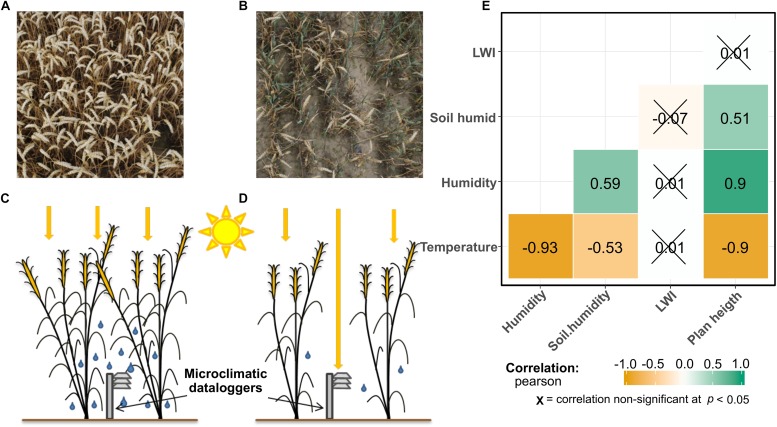
In panel **(A)** a bird view of a field point with high plant productivity, while **(B)** shows a point with a lower plant productivity. In panel **(C,D)**, a graphical representation of the canopy effect influencing the microclimatic data-loggers is shown. In more productive points **(C)**, the sunrays do not penetrate until the data-loggers, therefore the measured microclimate is colder and more humid, compared to less productive points **(D)**. Panel **(E)** shows a correlation matrix of the environmental variables. The numbers represent the Pearson coefficient (p) between them. Plotted using the package “ggstatsplot” in R (Patil, 2018).

Summer 2017 has been humid and rainy in the region of our study. In the month of June and July (when the study took place), we recorded a total rainfall of 141.1 and 129 mm, respectively. That is above the average for the region of Brandenburg, for which for the years between 1950 and 2016 has values of 61.8 mm in June and 68.2 mm in July (Deutscher Wetterdienst, Germany). An overview of the microclimatic data recorded within the field canopy is represented in the Supplementary Figure S2. Figure 2 shows how all the microclimatic conditions, besides LWI, are tightly interconnected. In Figure 2E, a correlation matrix of the microclimatic variables is shown. Air humidity and temperature show opposite trends, while soil humidity is less but significantly correlated to the other variables, but still showing significant correlations. Plant height is used as an indicator of field productivity. These values are explainable by considering the position of the microclimatic stations that were placed 30 cm above the ground under the plant canopy. At this height, plants shaded the sensors. Points with a higher productivity produce a higher shading effect, therefore reducing the average temperature and increasing air-humidity, while in less productive points the opposite trends happen. The results of microclimatic measurements show how air temperature, air humidity and plant height are tightly connected, with correlation coefficients same or higher than 0.9. Surprisingly, LWI does not show any correlations to the other measured parameters.

### Sequencing Results and Taxonomic Affiliation

**FIGURE 3:**
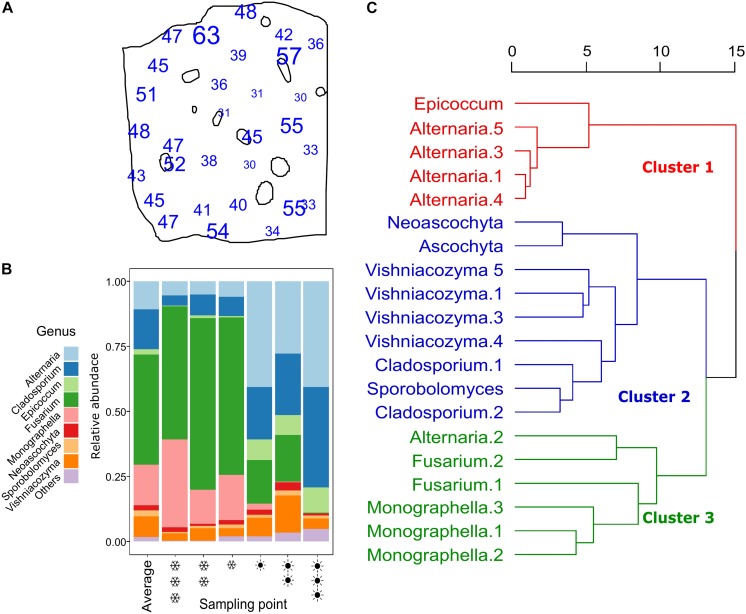
Panel **(A)** shows the number of different ASVs counted at each point. Panel **(B)** shows stacked barplots representing the community composition, where different colors symbolize different genera (only genera with a total abundance higher than 0.5% are shown, the remaining showed as “others”). The coldest point is represented by three 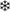 (snowflake) symbols, while the hottest by three 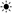 (sun). In panel **(C)**, a dendrogram representing the hierarchical cluster analysis obtained using Ward on a dissimilarity matrix of Euclidean distances of the 20 most abundant ASVs. The dendrogram is divided in three clusters, as suggested by the silhouette analysis. The three clusters are shown in different colors.

The ITS1 rRNA gene sequencing of all samples together generated 1,081,376 merged reads after quality checks, which resulted in 188 ASVs. Rarefaction curves showed that, for each sample, the method was adequate to sample the local diversity (Supplementary Figure S3). Of the 1,081,376 reads, 89% were classified as ascomycetes, 11% as basidiomycetes (the rest 0.02% were unclassified). The 188 ASVs were classified into 22 genera. In Figure 3A, the amount of detected ASVs per sampling point is represented on the plot map. On average, 43.2 ASVs were detected per sampling point. The point with the lowest number of detected ASVs had 30 ASVs, the one with the highest count had 63. The 20 most abundant ASVs counted for more than 95% of the total counts. Their taxonomic affiliation is reported in Table 1, with the two methods tested. The two methods to assign taxonomy produced concordant results, besides for the 2nd most abundant ASV, which was assigned by the UNITE database to “*Mycosphaerella tassiana*,” whereas, according to the blast on the genbank database, the sequence was affiliated to *Cladosporium* sp. In other cases, different species were assigned, but the genus was the same. For further analysis, we kept the assignments given by the GenBank blast search. *Cladosporium* was often reported as common colonizer of wheat ears, while *Mycosphaerella* are rather know to colonize ear leaves. The most abundant ASV was assigned to *Fusarium* sp., with 41% of relative abundance. All the others all showed a much lower abundance. The average composition of the whole field and the composition of the three hottest and warmest points are shown in Figure 3B. As we can see, in the three coldest points, the genus *Fusarium* and *Monographella* show a higher abundance compared to the average and the tree warmest points, whereas in the warmest points, *Cladosporium* and *Alternaria* represent the biggest fraction of the community.

**TABLE 1:** Taxonomic affiliation of the 20 most abundant ASVs, ranked according to their relative abundance.

**Relative abundance (%)**	**Dada2 assigned**	**Genbank BLAST**	**ID**
*41*	*Fusarium* sp.	*Fusarium* sp.	Fusarium.1
*13.3*	*Mycosphaerella tassiana*	*Cladosporium* sp.	Cladosporium.1
*9.5*	*Monographella nivalis*	*Monographella nivalis*	Monographella.1
*5.2*	*Alternaria infectoria*	*Alternaria infectoria*	Alternaria.1
*4.2*	*Vishniacozyma* sp.	*Vishniacozyma tephrensis*	Vishniacozyma.1
*2.9*	*Monographella nivalis*	*Monographella nivalis*	Monographella.2
*2.7*	*Monographella nivalis*	*Monographella nivalis*	Monographella.3
*2.2*	*Sporobolomyces roseus*	*Sporobolomyces roseus*	Sporobolomyces
*2.0*	*Cladosporium delicatulum*	*Cladosporium* sp.	Cladosporium.2
*2.0*	*Epicoccum nigrum*	*Epicoccum nigrum*	Epicoccum
*1.9*	*Alternaria infectoria*	*Alternaria infectoria*	Alternaria.2
*1.4*	*Vishniacozyma victoriae*	*Vishniacozyma victoriae*	Vishniacozyma.3
*1.3*	*Alternaria infectoria*	*Alternaria infectoria*	Alternaria.3
*1.3*	*Neoascochyta graminicola*	*Neoascochyta* sp.	Neoascochyta
*1.2*	*Vishniacozyma victoriae*	*Vishniacozyma victoriae*	Vishniacozyma.4
*0.7*	*Neoascochyta exitalis*	*Ascochyta skagwayensis*	*Ascochyta*
*0.7*	*Vishniacozyma victoriae*	*Vishniacozyma victoriae*	Vishniacozyma 5
*0.7*	*Alternaria infectoria*	*Alternaria infectoria*	Alternaria.4
*0.6*	*Fusarium lateritium*	*Fusarium* sp.	Fusarium.2
*0.5*	*Alternaria infectoria*	*Alternaria infectoria*	Alternaria.5

*Two methods were used for taxonomic affiliation, the first one was integrated in the dada2 R pipeline, the second one was based on a genbank BLAST search. The “ID” column identifies the names given to the different ASVs within this study.*

A clustering analysis was run to identify co-occurrence patterns of the 20 most abundant ASVs (Figure 3C). The clustering is based on the spatial distribution of the ASVs, therefore, ASVs showing a similar distribution, are clustered together. In the first cluster, four of the five *Alternaria* are present, together with *Epicoccum*. In cluster 2, yeasts (*Vishniacozyma* and *Sporobolomyces*) and filamentous fungi, specifically *Cladosporium*, *Neoascochyta* and *Ascochyta*. Cluster 3 has the genera *Fusarium* and *Monograhella*, with one *Alternaria* ASV also present.

### The Effect of Space

**FIGURE 4:**
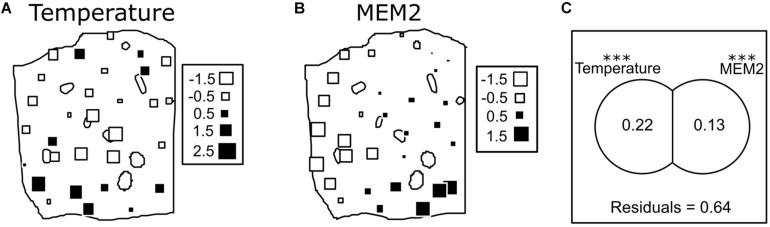
In panel **(A)** a map showing the variations of the measured under canopy temperature is shown. The measured values are standardized with 0 as the average and the standard deviation as unity. In panel **(B)** a map of the only Eigenvector map showing a statistically significant effect is shown (named MEM2, as the generated Eigenvector map having the second highest eigenvalue). The size and color of the square indicate the values of the eigenvector assigned at each point. Panel **(C)** is a visual representation of the variation partitioning between the two explanatory variables (or maps) used, it calculates the single explanatory power of the two matrixes and calculate an eventual joint effect. Due to a negative joint effect detected, the negative values were proportionally distributed between the two matrixes, as suggested by Legendre et al. (2012). The value within the circles are adjusted R squared. With residuals it is represented the fraction of unexplained variance (Signif. codes: ^∗∗∗^*p*-value < 0.001).

The raw ASV table was used as a base to calculate a Bray Curtis dissimilarity matrix. From the whole explanatory variables collected (air temperature, air humidity, soil humidity, plant height, and LWI), only air temperature, soil humidity, and LWI were kept for further analysis. Air humidity and plant height were dropped due to the high correlation coefficient they showed with air temperature, to avoid multicollinearity. These variables were tested as explanatory variables in a db-RDA using a Bray Curtis similarity matrix among point as response variable. Stepwise model building selected only temperature as explanatory variable, the spatial distribution of temperature in the field is represented in Figure 4A. To test for un-induced spatial variance, a Moran’s eigenvector maps (MEMs) based spatial analysis found one map showing a statistically significant correlation to our dataset. The map shows a positive spatial autocorrelation, therefore it was accepted as an explanatory spatial pattern. A total model was then built with these explanatory variables. Only one MEM, represented in Figure 4B, together with temperature, showed a significant effect in the final model. Variation partitioning was used to distinguish the explanatory power of the two variables identified the results present in Figure 4C. Both variables showed a statistically significant effect, with temperature explaining 25% of the variance (*F* = 11.10, *p* = *0.001*) and MEM explaining 15% of the variance (*F* = 5.7, *p* = *0.001*). Variables did not show any joint effect, therefore not showing any multicollinearity.

**FIGURE 5:**
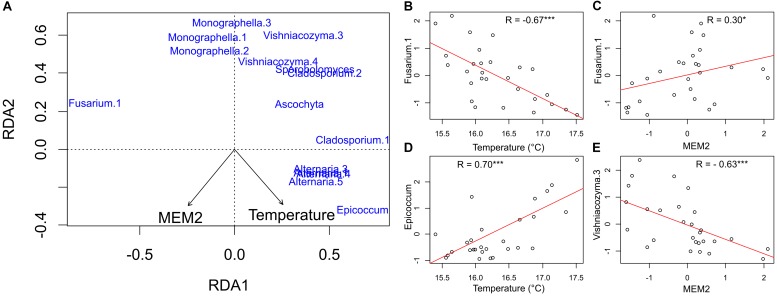
Panel **(A)** RDA ordination plot on standardized species data representing the community composition. As for ASVs, only the 20 most abundant ASVs with more than 20% of variance explained by the two constraining axes are shown. In panels **(B–E)** linear relationships between the ASVs counts and the explanatory variables are shown. Axes represent standardized values; therefore the unit is the group’s standard deviation (Signif. codes: ^∗∗∗^*p*-value < 0.001, ^∗^*p*-value < 0.05).

The RDA biplot shows the relationships between the different ASVs and the two significant explanatory variables (Figure 5). In Figure 5A, ASVs respond differently to the two variables. Fusarium.1 is the ASV showing the strongest negative correlation to temperature (*R* = −0.67, *p* < *0.0001*, Figure 5B), and positive to MEM2 (*R* = 0.32, *p* = *0.01*, Figure 5C). The strongest positive correlation to temperature was scored by Epicoccum (*R* = 0.70, *p* < *0.0001*, Figure 5D), while the strongest negative correlation to MEM2 was given by Vishniacozyma.3 (*R* = −0.63, *p* < *0.0001*, Figure 5E). Generally the 20 most abundant ASVs responded, beside Fusarium.1, negatively to the MEM map selected.

## Discussion

Through metabarcoding of the ITS1 segment, we showed how the phyllosphere fungal community can change within the same field due to spatial structures and differences in productivity. We identified 188 ASVs, nevertheless the 20 most abundant ones accounted for more than 95% of all counts. This confirms the results of previous studies done at a regional spatial scale, which reported a “core” mycobiome accompanied by rare strains only detected occasionally at lower abundances (Nicolaisen et al., 2014). Similar results were also observed on studies targeting cereal leaves, where a core of operational taxonomic units, ubiquitously present at high proportion was described (Karlsson et al., 2014, 2017; Sapkota et al., 2015, 2017). *Fusarium* dominated the community accounting for more than 40% of the total counts. The year 2017 was very rainy and conservation tillage was applied with wheat being cultivated after maize; factors known to promote the development of Fusarium head blight (Xu, 2003; Bateman et al., 2007; Manstretta and Rossi, 2015; Müller et al., 2016). If the field work was conducted in another year in a field with a different rotational history, the community composition would probably be consistently different.

The results of the db-RDA show how under canopy temperature, used as explanatory variable, is able to explain 25% of the total variance. Temperature measured at each point is directly connected to plant productivity, since the sensors were placed below the canopy cover, and measurements were strongly influenced by the abundance of leaves above the sensors. Points with a thicker canopy cover would be characterized by a lower temperature, while less productive points recorded a higher temperature. Therefore, plant productivity showed a significant effect on the microbial community across the field. The reason for such results might be different, and connected to a higher possibility for soil-borne pathogens to colonize the ear, given by the higher abundances of leaves, which might act as connecting “bridges” between the soil and the ear for spores carried by rain-splash (Xu, 2003; Manstretta et al., 2015; Schiro et al., 2018). A higher canopy cover effect, could also guarantee a better survival environment in the soil for the fungi, which might be less subject to temperature-humidity fluctuations and UV-light exposition, therefore having a higher amount of inoculum able to “climb up” to the ears once a rain event comes. These explanations are valid for those genera showing a negative correlation to temperature, such as *Fusarium* and *Monographella*. Genera like *Alternaria* and *Epicoccum* show a positive correlation to temperature; this might be connected to their host-preferences. They might find it easier to colonize plants in spots with a reduced productivity, since the plants are weaker and easier to colonize for saprotrophs. In this case it would be interesting to investigate the reasons for such differences in productivity. A link to water availability, given by different topographical position has been previously described (Müller et al., 2010). Nevertheless, plants can suffer from a variety of abiotic and biotic stresses, often acting at the same time (Suzuki et al., 2014). We speculate that a combination of factors, such as lower water availability or the action of soil pathogens, weaken the plant’s immune system, opening the path for the colonization of saprotrophic fungi, like members of the genera *Alternaria*, *Epicoccum* or *Cladosporium*. We did not find any study specifically addressing the effect of various plant stress conditions on the plant associated microbiota, although we are aware of the effort in trying to engineer plants microbiomes in order to enhance its stress resistance, such as drought (Coleman-Derr and Tringe, 2014; Ngumbi and Kloepper, 2016). This introduces a sort of cause-effect problem in the study of the microbiome of a plant: is the status of the plant influencing its microbiome, the microbiome regulating the status of the plant, or both phenomena are occurring at the same time? More efforts are needed to investigate what might be a sort of chained cause-effect loop.

We detected only one Eigenvector map showing a statistically significant correlation to our data. The db-RDA analysis, in this case, showed a smaller effect (15%) compared to temperature. The map shows the north-western part of the field having a different community then the south-eastern part, with all the most abundant ASVs, beside Fusarium.1, increasing toward the north-west. The reasons behind this geographical distribution are hard to predict. They could be the results of edge effects influencing the community, as the field was surrounded by different landscape components. While on the east and south side of the field there were other cultivated crops, in the west and north there were, respectively, a meadow and a forest edge. Fusarium.1 is a pathogen and its presence might be enhanced by the edges shared with other cultivated fields. On the other hand, yeasts like *Sporobolomyces* are described as naturally occurring saprophytic phyllosphere microorganisms; their presence might be boosted by the proximity to non-cultivated landscape elements, like a meadow and a forest edge in this case. Furthermore, Fusarium.1 is assigned either to *Fusarium graminearum* and/or *Fusarium culmorum;* these two species are described as very aggressive and their abundance might inhibit other fungal strains, like those showing a negative correlation to the MEM; a competitive interaction with *Monographella* was already described in the literature (Simpson et al., 2004). More efforts are necessary to clarify such patterns in relationship to the effect of different landscape structures and species interactions, studying their influence on the dispersal capacities of the members of the phyllosphere communities.

The clustering analysis of the obtained ASVs was based on their spatial distribution; ASVs with a similar distribution would show a smaller distance and therefore cluster together. The results here presented are similar results to those of Nicolaisen et al. (2014), who analyzed samples coming from different fields in Denmark (from a regional scale). Like this study, we obtained three main clusters. In cluster 1 *Epicoccum* and *Alternaria* are present, which can be classified as saprotrophs. Clusters 2 consists of yeasts and saprotrophs, while cluster 3 consists of fungi usually classified as pathogens (*Fusarium* and *Monographella*). Interestingly, Alternaria.2 is clustered in cluster 3, away from the other members of its genus. It displays a different spatial behavior than the other ASVs assigned to *Alternaria* in our studies. This fact introduces the need for having a self-made library for the taxonomic affiliation of our ASVs, where fungal isolates from the field will be characterized in order to better understand their behavior. This would be especially useful for the genus *Alternaria*, which complicated taxonomy is traditionally based on its morphology and sporulation patterns, but not always efficient in defining its biogeographical behavior or its aggressiveness toward the host plant. We selected ITS1 as metabarcoding marker; this method is already established and allowed us to have a comparison with previous studies (e.g., Nicolaisen et al., 2014). ITS1 is known to work well until genus level, but fails to identify species for important genera of fungi such as *Cladosporium* and *Fusarium* (Schoch et al., 2012). This is especially important for this study due to the high abundance of the genus *Fusarium*, where different species with different characteristics, e.g., *Fusarium graminearum* and *Fusarium culmorum*, cannot be distinguished. A powerful extension to our study, would involve the analysis of how different *Fusarium* species react to our explanatory variables, using a molecular approach able to reveal them (Boutigny et al., 2019).

To conclude, our results show how the phyllosphere fungal community changes within a topographically heterogeneous field. We show that these variations were related to potential explanatory variables, such as field productivity and autocorrelated spatial structures. In our case, field productivity, measured using under-canopy microclimatic variables, had a stronger explanatory effect than the geographical position of the sampling points. We also show how different fungal phyla behave differently in space, giving precious hints on their in-field epidemiology. We highlight that topographically heterogeneous fields are a valuable study system to investigate the ecology of phyllosphere fungi, showing similar dynamics observed in studies performed at a regional scale. With this study we also want to pinpoint the need for knowing more about the dispersal dynamics influencing the phyllosphere mycobiome, testing the effects of spatial structures at different scales and using different barcodes. Such observations are useful for the general challenge of understanding the drivers of wheat microbiome community assembly.

## Data Availability

Metabarcoding data is deposited as bioproject in the NCBI GenBank database (accession number: PRJNA517107).

## Author Contributions

GS and MM designed the study and conducted the field work. GS conducted the laboratory work and took the leadership in the writing of the manuscript. GS and PC analyzed the data. All authors contributed to the manuscript.

## Conflict of Interest Statement

The authors declare that the research was conducted in the absence of any commercial or financial relationships that could be construed as a potential conflict of interest.

## Footnotes

1 www.biomove.org/about-biomove/study_area/
