# Supplementary material for: A Metabarcoding Analysis of the Mycobiome of Wheat Ears Across a Topographically Heterogeneous Field
Source: Front Microbiol. 2019 Sep 10;10:2095. doi: 10.3389/fmicb.2019.02095 (PMC6746991; doi:10.3389/fmicb.2019.02095)
Supplement: Supplementary file 1 [file Data_Sheet_1.PDF]

# Tutorial, use of the R scripts provided with the article:

# "A Metabarcoding analysis of the mycobiome of wheat ears across a topographically heterogeneous field."

By Gabriele Schiro

For info contact me: gabschiro@gmail.com

How to repeat the data analysis:

First, unzip the content of the zip archive "scripts_and_data.zip".

The script "script_dada2.R" is the first script used, to go from the fastq files received by the sequencing providers, to the sequence count tab (usually known as OUT tab). This script was used as the bioinformatics pipeline. If you are interested in the statistical analysis, go directly to the "Instructions, statistical analysis" (paragraph 2). Both the scripts need some additional files (provided within the zip files), please leave these files in the same folder as the scripts.

## 1) Instructions, dada2 pipeline

This script was successfully run in a UNIX environment, using Ubuntu "18.04.1 LTS", R software version 3.5.1 and Rstudio.

1.1) Install "cutadapt" tool, following the instructions here provided:
https://cutadapt.readthedocs.io/en/stable/installation.html#quick-installation

1.2) The fastq files have been uploaded on the gen-bank database. We suggest to use the website of the European Nucleotide Archive: " https://www.ebi.ac.uk/", to download the starting fastq files.

1.3) Looking for the key: "PRJNA517107", will lead to the following webpage:

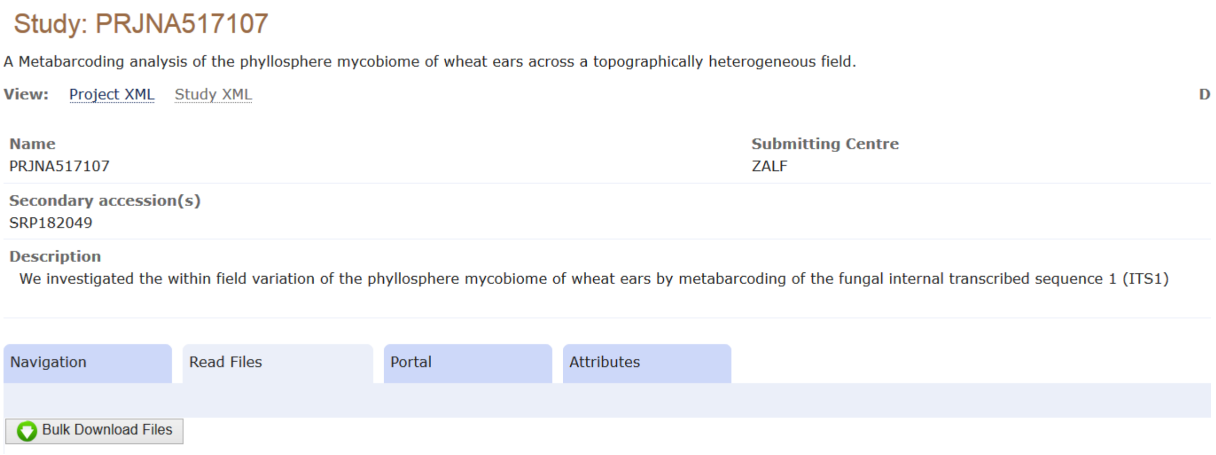


1.4) Pressing the button "bulk download files" will automatically download the compressed fastq (fastq.gz) files. These files can be then used inside the provided script called "script_dada2.R".

1.5) Open the file "script_dada2.R" with R (recommended R studio)

1.6) Insert the path leading to the folder where you have downloaded the fastq.gz files, in the line number 9 of the script:

```
path <- "/home/gabri/Desktop/fastq" ## CHANGE ME to the directory containing the fastq files.
```

1.7) Change the cutadapt path in the script (line number 62)

```
cutadapt <- "/home/gabri/.local/bin/cutadapt" # CHANGE ME to the cutadapt path on your machine
```

1.8) Download the UNITE reference database from the Unite website:

https://doi.org/10.15156/BIO/587475

1.9) Insert the path of the UNITE fasta file into line 149 of the script

```
unite.ref <-"file/sh_general_release_dynamic_s_01.12.2017.fasta"# CHANGE ME to location on your machine
```

1.10) Change the output paths of the outputfiles in the lines 153 and 154.

1.11) Run the script (beware, install all the R libraries necessary)

1.12) Once the script "script_dada2.R has terminated; it produces a file called seqtab.csv, which is the same as the one here provided, with different names of the sampling points. The taxonomic affiliations (tax.csv) are also outputted.

## 2) Instructions, statistical analysis

2.1) In this case you can directly run the R code "statistical_analysis.R" in R. Beware of the multitude of R libraries needed through the script, which might need previous installation.
