# Supplementary material for: A Metabarcoding Analysis of the Mycobiome of Wheat Ears Across a Topographically Heterogeneous Field
Source: Front Microbiol. 2019 Sep 10;10:2095. doi: 10.3389/fmicb.2019.02095 (PMC6746991; doi:10.3389/fmicb.2019.02095)
Supplement: Supplementary file 3 [file Data_Sheet_3.docx]

Supplementary Material

## Supplementary Table S1. Overview of the managing practices for the studied field.

| **Date** | **Product** | **Name** | **Concentration** | **Rates** | | |  |  |
| --- | --- | --- | --- | --- | --- | --- | --- | --- |
| 27.09.2016 | Sowing | Sort: Julius | 165 kg /ha |  | | |  |  |
| 16.03.2017 | Fertilizer | YaraBela Sulfan | 300 kg /ha | 73 kg N/ha, 18 kg Sa/ha | | |  |  |
| 27.03.2017 | Herbicide | Vertix | 0,083 kg /ha |  | | |  | |
| 05.04.2017 | Fertilizer | YaraBelaSulfan | 324 kg/ha | 78kg N/ha, 19 kg S/ha | | |  |  |
| 01.05.2017 | Plant growth regulator | Medax Top+ CCC | 0,078 + 720 l/ha | |  | |  |  |
| 01.05.2017 | Fungicide | Vegas + Cirkon | 0,236l/ha + 1,061 l/ha | | |  |  |  |
| 12.05.2017 | Fertilizer | Calcium ammonium nitrate | 150 kg /ha | | | 40,5 kg N/ha |  |  |
| 13.06.2017 | Fungizd | Taspa + Orius | 0,237l/ha + 0,947 l/ha | | |  |  |  |


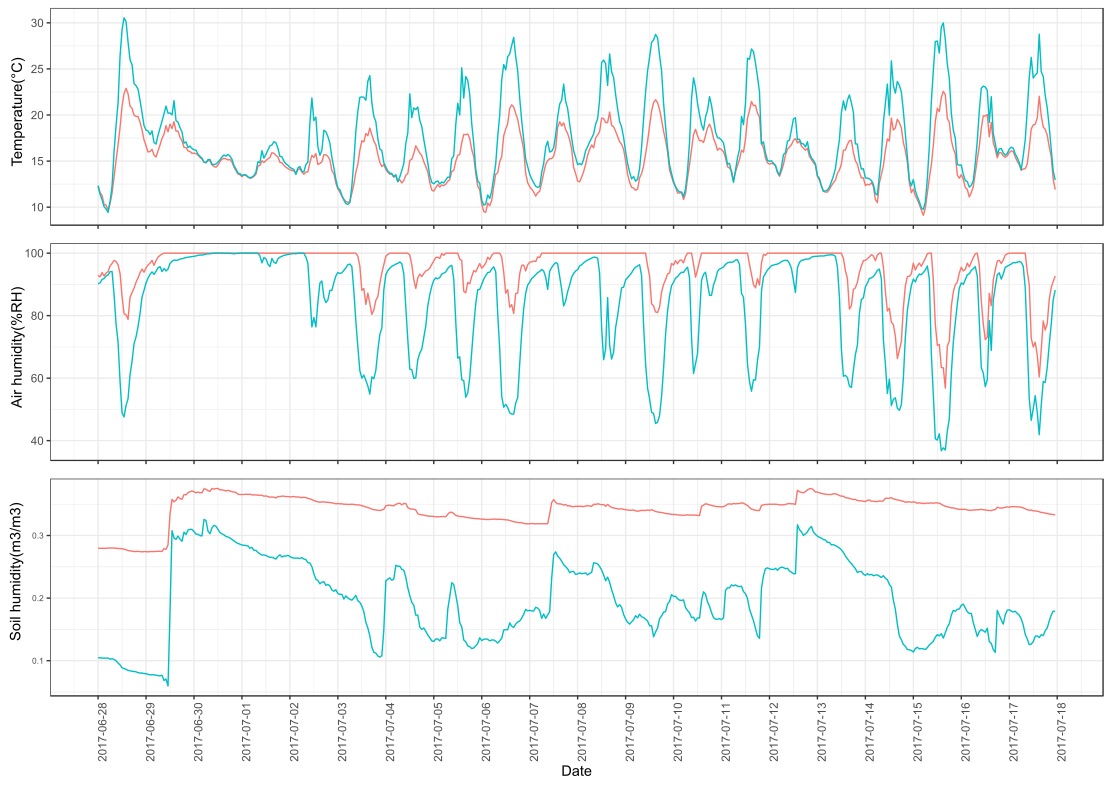


Supplementary Figure S1. : Overview of the microclimatic data recorded in the field, of the points with the most extreme microclimatic conditions


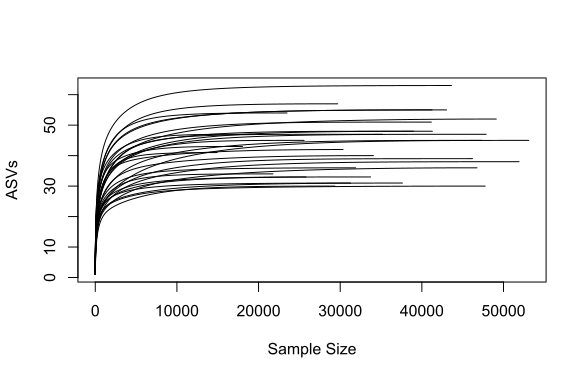


Supplementary Figure S2. Rarefaction curves of all the 30 sampling points in the field.
